# Antihyperglycemic and Antioxidative Effects of Hydroxyethyl Methylcellulose (HEMC) and Hydroxypropyl Methylcellulose (HPMC) in Mice Fed with a High Fat Diet

**DOI:** 10.3390/ijms13033738

**Published:** 2012-03-21

**Authors:** Su Jeong Ban, Catherine W. Rico, In Chul Um, Mi Young Kang

**Affiliations:** 1 Department of Food Science and Nutrition, Kyungpook National University, Daegu 702-701, Korea; E-Mails: crystal_hoi@nate.com (S.J.B.); ckwrico@gmail.com (C.W.R.); 2 Department of Natural Fiber Science, Kyungpook National University, Daegu 702-701, Korea; E-Mail: icum@knu.ac.kr

**Keywords:** hydroxyethyl methylcellulose, hydroxypropyl methylcellulose, glucose metabolism, antioxidative effect, high fat diet

## Abstract

The effect of dietary feeding of hydroxyethyl methylcellulose (HEMC) and hydroxypropyl methylcellulose (HPMC) on the glucose metabolism and antioxidative status in mice under high fat diet conditions was investigated. The mice were randomly divided and given experimental diets for six weeks: normal control (NC group), high fat (HF group), and high fat supplemented with either HEMC (HF+HEMC group) or HPMC (HF+HPMC group). At the end of the experimental period, the HF group exhibited markedly higher blood glucose and insulin levels as well as a higher erythrocyte lipid peroxidation rate relative to the control group. However, diet supplementation of HEMC and HPMC was found to counteract the high fat-induced hyperglycemia and oxidative stress via regulation of antioxidant and hepatic glucose-regulating enzyme activities. These findings illustrate that HEMC and HPMC were similarly effective in improving the glucose metabolism and antioxidant defense system in high fat-fed mice and they may be beneficial as functional biomaterials in the development of therapeutic agents against high fat dietinduced hyperglycemia and oxidative stress.

## 1. Introduction

Hydroxypropyl methyl cellulose (HPMC), a synthetically modified natural polymer, is a viscous soluble fiber widely used as a thickener, emulsifier, stabilizer, and gelling agent in the food and pharmaceutical industries [1]. Scientific studies have shown that it could lower the serum cholesterol content in hypercholesterolemic human subjects [2–5] and normalize metabolic abnormalities in obese mice [6]. It was also reported that consumption of HPMC significantly reduced postprandial glucose and insulin responses in overweight and obese men and women [7]. Like HPMC, the hydroxyethyl methylcellulose (HEMC) is also a methylcellulose derivative, but with ethyl group substitution. From an industrial viewpoint, HEMC can be produced more cheaply than HPMC, though functionally, they are similar. That is, HEMC is water soluble and a highly viscous material in the aqueous state like HPMC. However, studies on the functional properties of HEMC have been limited. While various researches have been conducted on the possible therapeutic potential of HPMC in the management of hypercholesterolemia and hyperglycemia, there have been no published reports yet on the physiological functions of HEMC.

Chronic consumption of a high fat diet leads to obesity, hyperlipidemia and hyperglycemia [8,9]. Studies have shown that individuals with higher dietary fat intake were more prone to develop glucose metabolism disorder and type 2 diabetes mellitus than those with lower fat intake [10]. The development of hyperglycemia is associated with oxidative stress resulting from increased generation of free radicals and reduced antioxidant defense mechanisms [11]. With the rapidly increasing incidence of diabetes worldwide, there is a greater need for safe and effective functional biomaterials with hypoglycemic activity and strong antioxidative effect.

Our recent investigation on HPMC and HEMC revealed that both of these dietary fibers were similarly effective in suppressing high fat-induced hyperlipidemia in mice [12]. Since HPMC has been previously reported to have an antihyperglycemic effect, it may be possible that HEMC also exhibits the same property. Hence, this study was conducted to determine whether HEMC, as well as HPMC, could improve the glucose metabolism in mice fed with a high fat diet. Furthermore, the comparative effects of these dietary fibers on the antioxidative defense system in mice were also investigated.

## 2. Results and Discussion

### 2.1. Body Weight Gain

At the end of the experimental period, the high fat (HF) group showed substantially higher body weight gain and amount of adipose tissues compared with the control group (Table 1). On the other hand, the HF+HEMC and HF+HPMC groups exhibited significantly lower body weight gain and amount of body fats relative to the HF group, despite the similar feed intake between the HF and HF+HPMC groups and the higher feed intake in the HF+HEMC group. This suggests that both HEMC and HPMC could control the high fat diet-induced body weight gain in mice.

### 2.2. Blood Glucose Level

**Figure 1:**
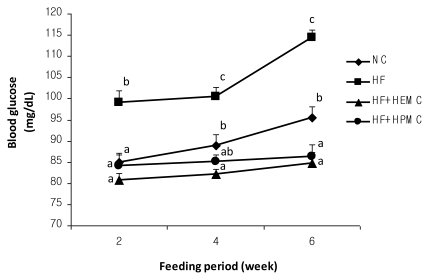
Effect of hydroxyethyl methylcellulose (HEMC) and hydroxypropyl methyl cellulose (HPMC) supplementation on the blood glucose level in high fat-fed mice. Means not sharing a common superscript are significantly different at *p* < 0.05 (n = 8). NC, normal control diet; HF, high fat diet; HF+HEMC, HF supplemented with HEMC; HF+HPMC, HF supplemented with HPMC.

After two weeks of feeding the mice with experimental diets, only the HF group exhibited a significant elevation in the blood glucose level (Figure 1). In the final week, the HF+HEMC and HPMC groups showed significantly lower glucose concentration than the NC and HF groups. An increasing trend in the glucose concentration through time was observed in all animal groups, which could probably be attributed to the aging of the mice. Armani *et al*. [13] reported that C57BL/6 mice fed with standard food pellets exhibited elevated blood glucose level from four to six weeks of age. Nonetheless, both HEMC and HPMC were able to suppress the increase in blood glucose level in mice. Previous studies have shown that diet supplementation with HPMC reduced the blood glucose concentrations in obese mice [6] hamsters [14], and human subjects [7]. This glucose-lowering effect of HPMC has been attributed to its high viscosity, a common property of soluble fibers [1,2]. When ingested, the HPMC forms a viscous solution in the gastrointestinal tract and acts as a barrier to the gastrointestinal absorption of glucose, thereby reducing the blood glucose concentrations [15,16]. Functionally, HPMC is very similar to HEMC. Their structure and properties are relatively the same. The present study demonstrated, for the first time, the hypoglycemic effect of HEMC, indicating that it may also be beneficial in the treatment and management of high fat diet-induced hyperglycemia.

### 2.3. Glycogen and Insulin Concentrations

The HF+HPMC group showed a marked increase in the glycogen concentration relative to the NC and HF groups (Table 2). Although not significant, an increase in the glycogen level was also observed in the HF+HEMC group. High fat feeding resulted in a substantial increase in the insulin concentration. However, supplementation of HEMC and HPMC in the diet significantly reduced the insulin level in mice relative to those fed with a high fat diet alone. Past studies have shown that methylcellulose can lower blood glucose level and increase the liver glycogen [17]. Hung *et al*. [6] also reported a decrease in the insulin level in mice fed with a high fat diet supplemented with HPMC. Moreover, consumption of high viscosity HPMC was shown to significantly blunt the postprandial glucose and insulin responses in obese men and women [18].

### 2.4. Plasma and Erythrocyte Lipid Peroxides

Oxidative stress, which results from increased generation of free radicals and an impaired antioxidant defense system, is associated with the development of hyperglycemia. High consumption of dietary fat has been shown to induce the formation of free radicals and reactive oxygen species, leading to lipid peroxidation and oxidative stress [19]. To assess the lipid peroxidation and oxidative stress in laboratory animals, analysis of the concentration of thiobarbituric acid reactive substances (TBARS) is widely employed. In the present study, a significant increase in the TBARS level in erythrocyte, but not in plasma, was observed in high fat-fed mice (Table 3). Conversely, diet supplementation of HEMC and HPMC suppressed the increased rate of lipid peroxidation, as evidenced by the lower plasma and erythrocyte TBARS concentration in the HF+HEMC and HF+HPMC groups relative to that of the HF and NC ones. Various dietary fibers, such as pectin, β-glucan, and blackgram fiber, have also been shown to decrease high fat diet-induced lipid peroxidation in animals [20,21]. Results of the present study provide the first evidence of the antioxidative effect of HEMC and HPMC, suggesting that these soluble dietary fibers may be helpful in preventing the progress of oxidative stress under high fat diet condition.

### 2.5. Hepatic Glucose-Regulating Enzyme Activities

Both the HF+HEMC and HF+HPMC groups showed significantly higher hepatic glucokinase (GK) activity than the NC and HF groups (Table 4). A substantial increase in glucose-6-phosphatase (G6pase) activity was observed in HF mice relative to the NC group. However, diet supplementation of HEMC and HPMC resulted in markedly reduced G6Pase activity in mice compared to those fed with a high fat diet alone. The HF+HEMC mice also exhibited significantly lower phosphoenolpyruvate carboxykinase (PEPCK) activity than the NC and HF ones. The enhancement in GK activity and inhibition of G6pase and PEPCK activities may have partly contributed to the hypoglycemic action of HEMC and HPMC. The hepatic GK enzyme is involved in the regulation of glucose homeostasis. An elevation in GK expression could cause an increase in blood glucose utilization for energy production or glycogen storage in the liver, leading to a decreased level of blood glucose [22]. The enhanced rate of glycogenesis observed in HEMC- and HPMC-fed mice, as manifested by an increase in hepatic glycogen level, was probably associated with the enhanced activity of GK enzyme. The hepatic G6pase and PEPCK enzymes, on the other hand, are involved in the regulation of gluconeogenesis and glucose output in the liver [23]. Thus, reduced activities of these enzymes indicate decreased hepatic glucose production.

### 2.6. Antioxidant Enzyme Activities

In order to regulate the destructive potential of free radicals and control oxidative stress, cells have developed a highly complex antioxidant protection system including antioxidant enzymes which catalyze free radical-quenching reactions. When the antioxidant system is impaired, the free radicals initiate lipid peroxidation and DNA damage, resulting in cell death [24]. In the present study, the activities of various antioxidant enzymes in the liver and erythrocyte were examined. Results showed that high fat feeding did not significantly change the activities of hepatic superoxide dismutase (SOD), catalase (CAT), paraoxonase (PON) and erythrocyte SOD, CAT, and glutathione reductase (GR) enzymes (Table 5). On the other hand, activities of these enzymes markedly increased in the HF+HEMC and HF+HPMC groups. The activity of hepatic GR significantly decreased in the HF group, but there was no significant change in the HF+HEMC and HF+HPMC groups, relative to that of the NC mice. The hepatic glutathione peroxidase (GSH-Px) activity was considerably higher in the control group than in those of the other groups, while the erythrocyte GSH-Px activity was lower in HF and HF+HPMC mice compared with that of the NC group. The decreased plasma and erythrocyte TBARS level and enhanced activities of SOD, CAT, and PON, and erythrocyte GR in HF+HEMC and HF+HPMC groups indicate a marked improvement in the *in vivo* antioxidative status of mice, making them less susceptible to peroxidative damage under a high fat diet condition. The SOD protects the cell from oxidative damage by converting superoxide radicals into hydrogen peroxides, which are then utilized and degraded by CAT and GSH-Px into non-toxic products [25]. The PON hydrolyzes biologically oxidized phospholipids and destroys lipid hydroperoxides [26], while the GR converts oxidized glutathione to antioxidant reduced glutathione [27]. The protective effect of HEMC and HPMC against high fat induced-lipid peroxidation and oxidative stress may partly be due to a mechanism involving the elevation of the activities of antioxidant enzymes. Moreover, the enhanced activity of SOD, accompanied by a decrease in GSH-Px activity, could have resulted in a higher intracellular concentration of hydrogen peroxides. Since hydrogen peroxide has an insulin-like effect [28], this increase in the intracellular level of peroxide, along with the enhanced activity of the GK enzyme, may have been partly responsible for the blood glucose-lowering action of HEMC and HPMC.

## 3. Experimental Section

### 3.1. Materials

The dietary fibers HEMC and HPMC were provided by Samsung Fine Chemicals (Ulsan, Korea). Their viscosities and degrees of substitution are shown in Table 6. All reagents were of analytical grade and used without further purification. The ketamine-HCl, ethanol, calcium chloride, tricholoroacetic acid, thiobarbituric acid, potassium phosphate buffer, and magnesium chloride were obtained from Merck KGaA (Darmstadt, Germany). All other chemicals were purchased from Sigma-Aldrich, Inc. (Steinhein, Germany).

### 3.2. Animals and Diet

Thirty-two male C57BL/6N mice of 4 weeks of age, weighing 12 g, were purchased from Orient Inc. (Seoul, Korea). They were individually housed in stainless steel cages in a room maintained at 25 °C with 50% relative humidity and 12/12 h light/dark cycle and fed with a pelletized chow diet for 2 weeks upon arrival. The animals were then randomly divided into 4 dietary groups (n = 8). The first and second mouse groups were fed with a normal control (NC) and a high fat (HF, 17%, w/w) diet, respectively. The other two groups were given a high fat diet supplemented with either HEMC (HF+HEMC) or HPMC (HF+HPMC). The composition of the experimental diet (Table 7) was based on the AIN-76 semisynthetic diet [29]. The mice were fed for 6 weeks and allowed free access to food and water during the experimental period. The food consumption and weight gain were measured daily and weekly, respectively. At the end of the experimental period, the mice were anaesthetized with ketamine-HCl following a 12-h fast. The blood samples were drawn from the inferior vena cava into a heparin-coated tube and centrifuged at 1,000 × g for 15 min at 4 °C to obtain the plasma and erythrocyte. The liver was removed, rinsed with physiological saline, and stored at −70 °C until analysis. The current study protocol was approved by the Ethics Committee of Kyungpook National University for animal studies.

### 3.3. Determination of Blood Glucose Level

The blood glucose level was measured using Accu-Chek Active Blood Glucose Test Strips (Roche Diagnostics GmbH, Germany). The blood samples were drawn from the tail vein of the mice after 2, 4, and 6 weeks of feeding the animals with experimental diets.

### 3.4. Measurement of Glycogen and Insulin Levels

The glycogen concentration was measured according to the method of Seifter *et al*. [30]. Fresh liver (100 mg) was mixed with 30% KOH and heated at 100 °C for 30 min. The mixture was added to 1.5 mL ethanol (95%) and kept overnight at 4 °C. The pellet was then mixed with 4 mL distilled water. A 500 μL sample of the mixture was added to 0.2% anthrone (in 95% H_2_SO_4_) and the absorbance of the sample solution was measured at 620 nm. The results were calculated on the basis of a standard calibration curve of glucose. The insulin content was measured using enzyme-linked immunosorbent assay (ELISA) kits (TMB Mouse Insulin ELISA kit, Sibayagi, Japan).

### 3.5. Determination of Lipid Peroxidation

The plasma and erythrocyte TBARS were determined using the method described by Ohkawa *et al*. [31]. Trichloroacetic acid (5%, v/v) and 0.06 M thiobarbituric acid were added to 50 μL of plasma and red blood cell preparation and incubated at 80 °C for 90 min. The mixtures were cooled to room temperature and centrifuged at 2,000 rpm for 25 min. The absorbance of the resulting supernatant was determined at 535 nm. A malondialdehyde (MDA) solution was used as standard and the results were calculated and expressed as nmol MDA/mL plasma or g Hb.

### 3.6. Determination of Hepatic Glucose-Regulating Enzyme Activities

The hepatic enzyme source was prepared according to the method described by Hulcher and Oleson [32]. The GK activity was determined based on the method of Davidson and Arion [33] with slight modification. A 0.98 mL sample of the reaction mixture containing 50 mM Hepes-NaOH (pH 7.4), 100 mM KCl, 7.5 mM MgCl_2_, 2.5 mM dithioerythritol, 10 mg/mL albumin, 10 mM glucose, 4 units of glucose-6-phosphate dehydrogenase, 50 mM NAD^+^ and 10 μL cytosol was pre-incubated at 37 °C for 10 min. The reaction was initiated by the addition of 10 μL of 5 mM ATP and the mixture was incubated at 37 °C for 10 min. The change in absorbance at 340 nm was recorded.

The G6pase activity was measured using the method of Alegre *et al*. [34]. The reaction mixture contained 765 μL of 131.58 mM Hepes-NaOH (pH 6.5), 100 μL of 18 mM EDTA (pH 6.5), 100 μL of 265 mM glucose-6-phosphate, 10 μL of 0.2 M NADP^+^, 0.6 IU/mL mutarotase and 0.6 IU/mL glucose dehydrogenase. After pre-incubation at 37 °C for 3 min, the mixture was added with 5 μL microsome and incubated at 37 °C for 4 min. The change in absorbance at 340 nm was measured.

The PEPCK activity was determined based on the method developed by Bentle and Lardy [35]. The reaction mixture consisted of 72.92 mM sodium Hepes (pH 7.0), 10 mM dithiothreitol, 500 mM NaHCO_3_, 10 mM MnCl_2_, 25 mM NADH, 100 mM IDP, 200 mM PEP, 7.2 units of malic dehydrogenase and 10 μL cytosol. The enzyme activity was determined based on the decrease in the absorbance of the mixture at 340 nm at 25 °C.

### 3.7. Measurement of Antioxidant Enzyme Activities

The SOD activity was spectrophotometrically measured based on the method of Marklund and Marklund [36]. The SOD was detected on the basis of its ability to inhibit superoxide-mediated reduction. The activity was expressed as unit/mg protein, wherein one unit represents the amount of enzyme that inhibits the oxidation of pyrogallol by 50%. The amount of protein was determined using Bradford protein assay [37].

The CAT activity was measured using the method of Aebi [38]. The disappearance of hydrogen peroxide was monitored spectrophotometrically at 240 nm for 5 min. A molar extinction coefficient of 0.041/mM/cm was used to determine the CAT activity. The activity was defined as the μmol decreased H_2_O_2_/min/mg protein.

The GSH-Px activity was measured according to the method of Paglia and Valentine [39] with slight modifications. The cytosolic supernatant was added to the reaction mixture (30 mM GSH, 6 mM NADPH, and 25 μM H_2_O_2_ in 0.1 mM Tris-HCl, pH 7.2), which was pre-warmed at 25 °C for 5 min. The mixture was further incubated at 25 °C for 5 min and the absorbance was measured at 340 nm. A molar extinction coefficient of 6.22/mM/cm was used to determine the activity, which was expressed as nmol oxidized NADPH/min/mg protein.

The GR activity was determined using the method of Mize and Langdon [40]. The reaction mixture contained 1 mM EDTA and 1 mM GSSG in a 0.1 M potassium phosphate buffer (pH 7.4). The oxidation of NADPH was monitored at 340 nm and the activity was expressed as nmol oxidized NADPH/min/mg protein.

The PON activity was determined based on the method described by Mackness *et al*. [41]. Briefly, 50 μL of serum was added to 1 mL Tris/HCl buffer (100 mM, pH 8.0) containing 2 mM CaCl_2_ and 5.5 mM paraoxon. The absorbance of the mixture was measured at 412 nm at 25 °C to determine the generation rate of 4-nitrophenol. The enzymatic activity was calculated using the molar extinction coefficient 17100/M/cm.

### 3.8. Statistical Analysis

All data are presented as the mean ± S.E. The data was evaluated by one-way ANOVA using a Statistical Package for Social Sciences software program (SPSS Inc., Chicago, IL, USA) and the differences between the means were assessed using Duncan’s multiple range test. Statistical significance was considered at *p* < 0.05.

## 4. Conclusions

Results of the study demonstrate that both HEMC and HPMC were effective in improving the blood glucose metabolism and suppressing oxidative stress in mice fed with a high fat diet. The antihyperglycemic and antioxidative effects of HEMC and HPMC could be partly attributed to the regulation of hepatic glucose-regulating enzyme activities and activation of the hepatic and erythrocyte antioxidant enzymes. HEMC and HPMC may be useful as biomaterials in the development of functional food or as therapeutic agents against high fat-induced hyperglycemia and oxidative stress.

## Figures and Tables

**Table 1:** Body weight gain, feed intake, and adipose tissue weight in mice fed with high fat diet supplemented with hydroxyethyl methylcellulose (HEMC) and hydroxypropyl methyl cellulose (HPMC).

	NC	HF	HF+HEMC	HF+HPMC
Body weight gain (g)	10.47 ± 0.72 ^a^	19.76 ± 0.36 ^d^	13.21 ± 0.83 ^b^	16.80 ± 0.61 ^c^
Total feed intake (g)	158.27 ± 3.12 ^a^	156.31 ± 2.03 ^a^	172.04 ± 4.63 ^b^	158.86 ± 2.99 ^a^
Adipose tissue weight (g)	7.62 ± 0.30 ^a^	12.02 ± 0.29 ^c^	10.30 ± 0.47 ^b^	10.27 ± 0.63 ^b^

Values are means ± SE (n = 8). Means in the same column not sharing a common superscript are significantly different at *p* < 0.05. NC, normal control diet; HF, high fat diet; HF+HEMC, HF supplemented with HEMC; HF+HPMC, HF supplemented with HPMC.

**Table 2:** Glycogen and insulin concentrations in mice fed with a high fat diet supplemented with HEMC and HPMC.

Dietary group	Glycogen (mg/g liver)	Insulin (ng/mL)
NC	4.87 ± 0.56 ^a^	4.83 ± 0.30 ^a^
HF	4.28 ± 0.44 ^a^	9.96 ± 0.42 ^c^
HF+HEMC	5.96 ± 0.94 ^ab^	6.40 ± 0.32 ^b^
HF+HPMC	6.76 ± 0.23 ^b^	6.82 ± 0.30 ^b^

Values are means ± SE (*n* = 8). Means in the same column not sharing a common superscript are significantly different at *p* < 0.05. NC, normal control diet; HF, high fat diet; HF+HEMC, HF supplemented with HEMC; HF+HPMC, HF supplemented with HPMC.

**Table 3:** Plasma and erythrocyte thiobarbituric acid reactive substances (TBARS) levels in mice fed with high fat diet supplemented with HEMC and HPMC.

Dietary group	Plasma TBARS (nmol/mL)	Erythrocyte TBARS (nmol/g Hb)
NC	13.76 ± 0.48 ^c^	4.95 ± 0.31 ^b^
HF	13.41 ± 0.34 ^bc^	6.59 ± 0.68 ^c^
HF+HEMC	10.86 ± 0.30 ^a^	3.57 ± 0.02 ^a^
HF+HPMC	12.82 ± 0.16 ^b^	3.46 ± 0.19 ^a^

Values are means ± SE (*n* = 8). Means in the same column not sharing a common superscript are significantly different at *p* < 0.05. NC, normal control diet; HF, high fat diet; HF+HEMC, HF supplemented with HEMC; HF+HPMC, HF supplemented with HPMC.

**Table 4:** Hepatic glucose-regulating enzyme activity in mice fed with a high fat diet supplemented with HEMC and HPMC.

	Enzyme activity (nmol/min/mg protein)		
	
Dietary group	GK	G6pase	PEPCK
NC	2.09 ± 0.14 ^a^	162.00 ± 7.33 ^a^	3.16 ± 0.31 ^b^
HF	2.13 ± 0.06 ^a^	287.00 ± 13.00 ^c^	3.49 ± 0.14 ^b^
HF+HEMC	3.64 ± 0.07 ^b^	214.44 ± 7.39 ^b^	2.06 ± 0.51 ^a^
HF+HPMC	3.97 ± 0.14 ^c^	218.55 ± 8.53 ^b^	2.65 ± 0.23 ^ab^

Values are means ± SE (*n* = 8). Means in the same column not sharing a common superscript are significantly different at *p* < 0.05. NC, normal control diet; HF, high fat diet; HF+HEMC, HF supplemented with HEMC; HF+HPMC, HF supplemented with HPMC. Glucokinase = GK, phosphoenolpyruvate carboxykinase = PEPCK, glucose-6-phosphatase = G6pase.

**Table 5:** Antioxidant enzyme activity in mice fed with high fat diet supplemented with HEMC and HPMC.

	NC	HF	HF+HEMC	HF+HPMC
*Liver*				
SOD (unit/mg protein)	1.56 ± 0.14 ^a^	1.32 ± 0.12 ^a^	2.59 ± 0.15 ^b^	2.31 ± 0.25 ^b^
GSH-Px (nmol/min/mg protein)	4.03 ± 0.13 ^c^	3.75 ± 0.05 ^b^	3.14 ± 0.11 ^a^	3.30 ± 0.08 ^a^
CAT (μmol/min/mg protein)	1.17 ± 0.03 ^a^	1.14 ± 0.01 ^a^	1.46 ± 0.02 ^b^	1.49 ± 0.01 ^b^
GR (nmol/min/mg protein)	16.13 ± 0.67 ^b^	12.82 ± 1.00 ^a^	14.57 ± 0.99 ^ab^	14.74 ± 0.79 ^ab^
PON (nmol/min/mg protein)	2.48 ± 0.30 ^a^	2.26 ± 0.14 ^a^	3.85 ± 0.33 ^b^	3.86 ± 0.58 ^b^
*Erythrocyte*				
SOD (unit/mg Hb)	4.87 ± 0.33 ^a^	4.35 ± 0.13 ^a^	7.85 ± 1.02 ^b^	8.25 ± 1.00 ^b^
GSH-Px (nmol/min/g Hb)	2.15 ± 0.15 ^b^	1.62 ± 0.05 ^a^	1.97 ± 0.23 ^ab^	1.70 ± 0.05 ^a^
CAT (μmol/min/g Hb)	0.37 ± 0.01 ^a^	0.33 ± 0.01 ^a^	0.48 ± 0.04 ^b^	0.51 ± 0.02 ^b^
GR (μmol/min/g Hb)	0.27 ± 0.02 ^a^	0.27 ± 0.01 ^a^	0.82 ± 0.16 ^b^	0.73 ± 0.17 ^b^

Values are means ± SE (*n* = 8). Means in the same row not sharing a common superscript are significantly different at *p* < 0.05. NC, normal control diet; HF, high fat diet; HF+HEMC, HF supplemented with HEMC; HF+HPMC, HF supplemented with HPMC. Hepatic superoxide dismutase (SOD), catalase (CAT), and paraoxonase (PON), glutathione peroxidase (GSH-Px), glutathione reductase (GR).

**Table 6:** Viscosity and degree of substitution in HEMC and HPMC.

Sample	Viscosity (cps)	Degree of substitution (%)		
		
		Methyl group	Ethyl group	Propyl group
HEMC	53900	28.30	8.34	-
HPMC	49000	22.21	-	8.68

**Table 7:** Composition of the experimental diets (%).

Component	Dietary group			

	NC	HF	HF+HEMC	HF+HPMC
Casein	20.0	20.0	20.0	20.0
DL-Methionine	0.3	0.3	0.3	0.3
Sucrose	50.0	50.0	48.0	48.0
Cellulose	5.0	5.0	5.0	5.0
Corn oil	5.0	3.0	3.0	3.0
Choline bitartrate	0.2	0.2	0.2	0.2
Mineral mixture a	3.5	3.5	3.5	3.5
Vitamin mixture b	1.0	1.0	1.0	1.0
Corn starch	15.0	-	-	-
Lard	-	17.0	17.0	17.0
HEMC	-	-	2.0	-
HPMC	-	-	-	2.0
Total	100.0	100.0	100.0	100.0

NC, normal control diet; HF, high fat diet; HF+HEMC, HF supplemented with HEMC; HF+HPMC, HF supplemented with HPMC

a AIN-76 mineral mixture;

b AIN-76 vitamin mixture.

## References

[1] Burdock G.A. (2007). Safety assessment of hydroxypropyl methylcellulose as a food ingredient. Food Chem. Toxicol.

[2] Maki K.C., Carson M.L., Anderson W.H.K., Geohas J., Reeves M.S., Farmer M.V., Turowski M., Miller M., Kaden V.N., Dicklin M.R. (2009). Lipid-altering effects of different formulations of hydroxymethylcellulose. J. Clin. Lipidol.

[3] Maki K.C., Davidson M.H., Malik K.C., Albrecht H.H., O’Mullane J., Daggy B.P. (1999). Cholesterol lowering with high-viscosity hydroxypropylmethylcellulose. Am. J. Cardiol.

[4] Reppas C., Swidan S.Z., Tobey S.W., Turowski M., Dressman J.B. (2009). Hydroxypropylmethylcellulose significantly lowers blood cholesterol in mildly hypercholesterolemic human subjects. Eur. J. Clin. Nutr.

[5] Swidan S.Z., Reppas C., Barnett J.L., Grenwood D.E., Tallman A.M., Tobey S.W., Dressman J.B. (1996). Ability of two comestible formulations of hydroxypropylmethylcellulose to lower serum cholesterol concentrations. Eur. J. Pharm. Sci.

[6] Hung S.C., Anderson W.H.K., Albers D.R., Langhorst M.L., Young S.A. (2011). Effect of hydroxypropyl methylcellulose on obesity and glucose metabolism in a diet-induced obesity mouse model. J. Diabetes.

[7] Maki K.C., Carson M.L., Miller M.P., Turowski M., Bell M., Wilder D.M., Rains T.M., Reeves M.S. (2008). Hydroxypropylmethylcellulose and methylcellulose consumption reduce postprandial insulinemia in overweight and obese men and women. J. Nutr.

[8] Bray G.A., Paeratakul S., Popkin B.M. (2004). Dietary fat and obesity: A review of animal, clinical and epidemiological studies. Physiol. Behav.

[9] Park J., Rho H.K., Kim K.H., Choi S.S., Lee Y.S., Kim J.B. (2005). Overexpression of glucose-6-phosphate dehydrogenase is associated with lipid dysregulation and insulin resistance in obesity. Mol. Cell. Biol.

[10] Lichtenstein A.H., Schwab U.S. (2000). Relationship of dietary fat to glucose metabolism. Atherosclerosis.

[11] Sharma N., Garg V., Paul A. (2010). Antihyperglycemic, antihyperlipidemic and antioxidative potential of *Prosopis cineraria* bark. Indian J. Clin. Biochem.

[12] Ban S.J., Rico C.W., Um I.C., Kang M.Y. (2012). Comparative evaluation of the hypolipidemic effects of hydroxyethyl methylcellulose (HEMC) and hydroxypropyl methylcellulose (HPMC) in high fat-fed mice. Food Chem. Toxicol.

[13] Amrani A., Durant S., Throsby M., Coulaud J., Dardenne M., Homo-Delarche F. (1998). Glucose homeostasis in the nonobese diabetic mouse at the prediabetic stage. Endoctrinology.

[14] Hung S.C., Bartley G., Young S.A., Albers D.R., Dielman D.R., Anderson W.H.K., Yokohama W. (2009). Dietary fiber improves lipid homeostasis and modulates adipocytokines in hamsters. J. Diabetes.

[15] Reppas C., Adair C.H., Barnett J.L., Berardi R.R., DuRoss D., Swidan S.Z., Thill P.F., Tobey S.W., Dressman J.B. (1993). High viscosity hydroxypropylmethylcellulose reduces postprandial blood glucose concentrations in NIDDM patients. Diabetes Res. Clin. Pract.

[16] Reppas C., Greenwood D.E., Dressman J.B. (1999). Longitudinal versus radial effects of hydroxypropyl methylcellulose on gastrointestinal glucose absorption in dogs. Eur. J. Pharmacol. Sci.

[17] Topping D.L., Oakenfull D., Trimble R.P., Illman R.J. (1988). A viscous fibre (methylcellulose) lowers blood glucose and plasma triacylglycerols and increases liver glycogen independently of volatile fatty acid production in the rat. Br. J. Nutr.

[18] Maki K.C., Carson M.L., Miller M.P., Turowski M., Bell M., Wilder D.M., Reeves M.S. (2007). High-viscosity hydroxypropylmethylcellulose blunts postprandial glucose and insulin responses. Diabetes Care.

[19] Ibrahim W., Lee U.S., Yeh C.C., Szabo J., Bruckner G., Chow C.K. (1997). Oxidative stress and antioxidant status in mouse liver: Effects of dietary lipid, vitamin E and iron. J. Nutr.

[20] Sanchez D., Quiñones M., Moulay L., Muguerza B., Miguel M., Aleixandre A. (2011). Soluble fiber-enriched diets improve inflammation and oxidative stress biomarkers in Zucker fatty rats. Pharmacol. Res.

[21] Thampi B.S., Manoj G., Leelamma S., Menon V.P. (1991). Dietary fiber and lipid peroxidation: Effect of dietary fiber on levels of lipids and lipid peroxides in high fat diet. Indian J. Exp. Biol.

[22] Coope G.J., Atkinson A.M., Allott C., McKerrecher D., Johnstone C., Pike K.G., Holme P.C., Vertigan H., Gill D., Coghlan M.P. (2006). Predictive blood glucose lowering efficacy by glucokinase activators in high fat fed female zucker rats. Br. J. Pharmacol.

[23] Friedman J.E., Sun Y., Ishizuka T., Farrell C.J., McCormack S.E., Herron L.M., Hakimi P., Lechner P., Yun J.S. (1997). Phosphoenolpyruvate carboxykinase (GTP) gene transcription and hyperglycemia are regulated by glucocorticoids in genetically obese db/db transgenic mice. J. Biol. Chem.

[24] Devi G.S., Prasad M.H., Saraswathi I., Raghu D., Rao D.N., Reddy P.P. (2000). Free radicals antioxidant enzymes and lipid peroxidation in different types of leukemia. Clin. Chim. Acta.

[25] Reiter R.J., Tan D., Burkhardt S. (2002). Reactive oxygen and nitrogen species and cellular and organismal decline:amelioration with melatonin. Mech. Aging Dev.

[26] Ng C.J., Shih D.M., Hama S.Y., Villa N., Navab M., Reddy S.T. (2005). The paraoxonase gene family and atherosclerosis. Free Radic. Biol. Med.

[27] Mullineaux P.M., Creissen G.P., Scandalios J.G. (1997). Glutathione Reductase: Regulation and Role in Oxidative Stress. Oxidative Stress and the Molecular Biology of Antioxidant Defenses.

[28] May S., de Haen C. (1979). The insulin-like effect of hydrogen peroxide on pathways of lipid synthesis in rat adipocytes. J. Biol. Chem.

[29] American Institute of Nutrition (1980). Report of ad hoc committee on standards for nutritional studies. J. Nutr..

[30] Seifter S., Dayton S., Navic B., Muntwyler E. (1950). The estimation of glycogen with the anthrone reagent. Arch. Biochem.

[31] Ohkawa H., Ohishi N., Yagi K. (1979). Assay for lipid peroxides in animal tissues by thiobarbituric acid reaction. Anal. Biochem.

[32] Hulcher F.H., Oleson W.H. (1973). Simplified spectrophotometric assay for microsomal 3-hydroxy-3-methylglutaryl CoA reductase by measurement of coenzyme A. J. Lipid Res.

[33] Davidson A.L., Arion W.J. (1987). Factors underlying significant underestimations of glucokinase activity in crude liver extracts: Physiological implications of higher cellular activity. Arch. Biochem. Biophys.

[34] Alegre M., Ciudad C.J., Fillat C., Guinovart J.J. (1988). Determination of glucose-6-phosphatase activity using the glucose dehydrogenase-coupled reaction. Anal. Biochem.

[35] Bentle L.A., Lardy H.A. (1976). Interaction of anions and divalent metal ions with phosphoenolpyruvate carboxykinase. J. Biol. Chem.

[36] Marklund S., Marklund G. (1974). Involvement of the superoxide anion radical in the autoxidation of pyrogallol and convenient assay for superoxide dismutase. Eur. J. Biochem.

[37] Bradford M.M. (1976). A rapid sensitive method for the quantitation of microgram quantities of protein utilizing the principle of protein-dye binding. Anal. Biochem.

[38] Aebi H, Bergmeyer H.U. (1974). Catalase. Method of Enzymatic Analysis.

[39] Paglia E.D., Valentine W.N. (1967). Studies on quantitative and qualitative characterization of erythrocyte glutathione peroxidase. J. Lab. Clin. Med.

[40] Mize C.E., Langdon R.G. (1952). Hepatic glutathione reductase, purification and general kinetic properties. J. Biol. Chem.

[41] Mackness M.I., Arrol S., Durrington P.N. (1991). Paraoxonase prevents accumulation of lipoperoxides in low-density lipoprotein. FEBS Lett.

